# The Moquis

**Published:** 1881-05

**Authors:** 


### THE MOQUIS.

In the history of the aboriginal races of this country, little is said regarding the Moquis, a branch of the Pueblos, living where possibly they have lived for a thousand years, in a rocky stronghold in a sandy desert of Arizona. This people number about two thousand five hundred, and occupy six villages, with houses built of stone, cemented with sand and clay. These villages, says Dr. Loew, of Wheeler's surveying expedition, are built on the tops of four sandstone mesas, which are separated from each other about eight miles. They occupy the entire width of the mesas, and, standing immediately before the houses, one may look vertically down a depth of three hundred feet. In many places the sides of the mesas are terraced, being used as sheep corrals. In appearance the Moquis come rather nearer to the Caucasian than the rest of his race. These

Indians are well clad, and the females especially so. Indian corn is the principal food—the sheep are raised for their wool rather than for the table. From the wool a good blanket is made. The seed corn is planted about one and a-half feet from the surface, at which depth sufficient moisture is found to develope and sustain the plant. The Moquis have neither church or any other place of worship, and the Spanish Jesuits were unable to gain a foothold among them.—*Kansas City Review.*

